# The phylogenetic, pathogenicity and transmission capacity analysis of one H9N2 strain

**DOI:** 10.3389/fvets.2025.1639235

**Published:** 2025-12-24

**Authors:** LiJia Meng, XinHao Tang, WenYuan Gu, Cheng Zhang, Shishan Dong

**Affiliations:** 1 College of Veterinary Medicine Traditional Chinese Veterinary Medicine, Hebei Agricultural University, Baoding, China; 2 Shijiazhuang Shengbo Biological Technology Co., Ltd., Shijiazhuang, China; 3 Hebei Provincial Center for Animal Diseases Control and Prevention, Shijiazhuang, China

**Keywords:** phylogenetic analysis, H9-subtype, avian influenza viruses, pathogenicity, transmission capacity

## Abstract

Avian influenza is an acute and highly contagious infectious disease that can infect multiple hosts. The low pathogenic avian influenza virus (LP-AIV), represented by the H9-subtype can cause a decrease in egg production and immunosuppression, lead to respiratory diseases and other diseases, reduce poultry production efficiency, and seriously affect the sustainable development of the poultry industry. In this study, one strain of H9-subtype AIV was isolated, purified, and named A/Hebei/723/2019 (H9N2). Phylogenetic analysis indicated that the HA, NA and M genes of the isolated strain belonged to the Y280-like lineage in the Eurasian lineage. The PA, NP and NS genes belong to the SH/F/98-like lineage. The PB1 gene belongs to the Y439-like lineage. The PB2 gene belongs to the G1-like lineage. Moreover, this strain binds exclusively to the *α*-2,6 sialic acid receptor, exhibits low pathogenicity to mice, and can be transmitted directly through contact and aerosol transmission among guinea pigs. It has a potential risk of infecting mammals and provides reference value for the subsequent formulation of measures for disease prevention and control.

## Highlights

- The genetic evolution of surface gene and internal gene coding sequences of a strain named A/Hebei/723/2019 (H9N2) were analyzed.
- The isolated and purified strain has relatively weak pathogenicity to mice.
- The strain can be directly contacted and transmitted through aerosols among guinea pigs, posing a potential risk of infecting mammals.

## 1 Introduction

Avian influenza is an acute and highly contagious infectious disease that can infect a variety of hosts, including poultry, resident birds, migratory birds, and some mammals (1–3). The pathogen is avian influenza viruses (AIV), which consist of eight independent single-stranded negative-stranded RNA (vRNA) and belong to the genus *Influenza A* of the *Orthomyxoviridae* family (4, 5). AIV has a variety of subtypes, and there is no cross protection between subtypes, and the antigen variation is frequent (6). Based on their pathogenicity to poultry, AIV can be divided into highly pathogenic avian influenza viruses (HP-AIV) and low pathogenic avian influenza viruses (LP-AIV) (4). HP-AIV, represented by the H5 and H7 subtypes, spread quickly, are highly virulent, and can cause acute, fatal diseases in poultry. Moreover, there have been reports of human infections with subtypes such as H5N1 and H7N9 (7, 8). LP-AIV can lead to a decline in egg production, immunosuppression, respiratory and other diseases, reduce poultry production efficiency, and serious threats to the sustainable development of the poultry industry. Among them, the H9N2 subtype is particularly important to public health, as it is not only widespread globally but also capable of crossing species barriers to infect humans (9, 10).

The H9-subtype is the most widespread subtype of AIV in poultry in Asia and has potential pandemic risk. Since the beginning of the epidemic of H9-subtype AIV in China (11, 12), the vaccine strains targeting H9-subtype AIV have been widely used (13–15), but there are still persistent clinical infections in chickens, and even immunized chickens can be infected, leading to outbreaks of the epidemic (16). In addition, the H9-subtype AIV is prone to antigenic mutations during epidemics and may recombine with other subtype strains. For example, in recent years, in the newly discovered H10N8 and H5N6 subtypes that can infect humans, some of the internal gene segments have been found to come from the H9-subtype (17–19). Although the H9-subtype AIV belongs to LP-AIV, it can cause severe respiratory symptoms by reducing the body’s immune response to secondary infections of viruses or bacteria, causing heavy losses to the poultry industry, and posing a serious threat to human health and biosecurity (20, 21).

Although H9-subtype AIV has already been reported in China (11, 22, 23), knowledge about effective control measures against this infectious disease is limited. Our study conducted a genetic evolution analysis of the surface and internal gene coding sequences of an isolated and purified H9-subtype AIV strain. We also investigated its pathogenicity and transmission patterns in mice, aiming to gain a deeper understanding of the virus’s biological characteristics in a mammalian model. These findings provide a scientific basis for assessing its public health risks and formulating prevention and control strategies.

## 2 Materials and methods

### 2.1 Virus isolation and purification

Through conventional molecular identification and virus isolation, one strain of H9 subtype avian influenza virus was successfully isolated from the ground wastewater of a closed chicken house in Baoding City, Hebei Province, in 2019. It was purified and named A/Hebei/723/2019 (H9N2).

The supernatant obtained from H9-subtype positive samples after pretreatment was filtered and sterilized with a 0.22 μm filter. It was then inoculated into 9-day-old SPF chicken embryos through the allantoic cavity, incubated at 37 °C for 48 h. The allantoic fluid was harvested and the hemagglutination value was determined as described previously.

Chicken embryo limited dilution method: The virus containing allantoic fluid was serially diluted tenfold from 10^−5^ to 10^−9^. A volume of 0.2 mL from each dilution was inoculated into individual embryos. The allantoic fluid with the highest hemagglutination titer was harvested, and the dilution and purification process was repeated for subsequent rounds. After three consecutive rounds of purification, the supernatant was obtained by centrifugation at 3000 rpm for 10 min at 4 °C, aliquoted, and stored at −80 °C.

### 2.2 Genome sequencing

To analyze the genetic divergence of H9-subtype AIV isolated in this study, the viral RNA was extracted from allantoic fluid using a DNA/RNA extraction kit (TIANGEN, Beijing, China) following the manufacturer’s instructions and eluted with 20 μL RNase-free water. The surface and internal genes coding sequences were amplified by RT-PCR using the PrimeScript^™^ One Step RT-PCR Kit (Code No. RR055A, TaKaRa, China) according to the manufacturer’s instructions. The reaction mixture (50.0 μL) comprised 25.0 μL of 2 × 1 Step Buffer, 2.0 μL of PrimeScript 1 Step Enzyme Mix, 1.0 μL of each primer pair, 19.0 μL of nuclease-free water and 2.0 μL of nucleic acid template. Thermal cycling conditions involved an initial 30 min incubation at 50 °C, then 94 °C for 2 min, followed by 30 cycles of 98 °C for 10 s and 68 °C for 1 min. Specific primers used in this study are listed in Table 1. The PCR products were purified and sequenced (Tsingke Biotechnology, China). M13F (*TGTAAAACGACGGCCAGT*) or M13R (*CAGGAAACAGCTATGACC*) sequence (black italics) was introduced at the 5′ end of the primer.

**Table 1:** RT-PCR primers used in this study.

Primer name	Primer sequence (5′-3′)
HA-F	*TGTAAAACGACGGCCAGT*AGCAAAAGCAGGGGAAT
HA-R	*CAGGAAACAGCTATGACC*AGTAGAAAACAAGGGTGT
NA-F	*TGTAAAACGACGGCCAGT*AGCAAAAGCAGGAGTA
NA-R	*CAGGAAACAGCTATGACC*AGTAGAAACAAGGAGTTT
PB2-F	*TGTAAAACGACGGCCAGT*AGCAAAAGCAGGTCAATT
PB2-R	*CAGGAAACAGCTATGACC*AGTAGAAACAAGGT
PB1-F	*TGTAAAACGACGGCCAGT*AGCGAAAGCAGGCAAACCAT
PB1-R	*CAGGAAACAGCTATGACC*AGTAGAAACAAGGCATTT
PA-F	*TGTAAAACGACGGCCAGT*AGCAAAAGCAGGTACT
PA-R	*CAGGAAACAGCTATGACC*AGTAGAAACAAGGTACTTT
NP-F	*TGTAAAACGACGGCCAGT*AGCAAAAGCAGGGT
NP-R	*CAGGAAACAGCTATGACC*AGTAGAAACAAG
M-F	*TGTAAAACGACGGCCAGT*AGCAAAAGCAGGTAGATAT
M-R	*CAGGAAACAGCTATGACC*AGTAGAAACAAGGTAGTT
NS-F	*TGTAAAACGACGGCCAGT*AGCAAAAGCAGGGTGACA
NS-R	*CAGGAAACAGCTATGACC*AGTAGAAACAAGGGTGTT

### 2.3 Genetic and phylogenetic analyses

In order to better understand the H9-subtype AIV strain’s genetic characteristics, the H9-subtype AIV sequences obtained in our study, along with the reference strains (including BJ/94-like strains, Y280-like strains, G1-like strains, SH/F/98-like strains, Y439-like strains, and North American strains) from various regions downloaded from the NCBI nucleotide database, were aligned using Lasergene software with the Clustal W program for homology analysis. The phylogenetic trees of the surface genes and internal genes coding sequences (for HA, NA, PB2, PB1, PA, NP, M, NS) were generated using MEGA11 software with the neighbor-joining algorithm and bootstrap analysis with 1,000 replicates. Genome sequence alignments of the surface genes and internal genes were analyzed using BioAider V1.423.

### 2.4 The receptor binding characteristics

The strain was serially diluted in PBS buffer using a 2-fold gradient to a final concentration of 2^−10^. Then, 1% chicken red blood cells (cRBC), 1% sheep red blood cells (sRBC), cRBC containing *α*-2, 3-sialidase, and cRBC treated with 50 mU/μL *Vibrio cholerae* neuraminidase (VCNA enzyme) were each added to 96-well hemagglutination plates. The mixtures were thoroughly mixed and incubated at 37 °C for 15–20 min. The hemagglutination titer of the strain was determined by observing the agglutination of red blood cells, and the ability of the strain to bind to the avian *α*-2,3 receptor and the human α-2,6 receptor was investigated.

### 2.5 The growth dynamic curves of the strain on chicken embryos and MDCK cells

The diluted virus solution was inoculated into 9-day-old SPF chicken embryos. At 12, 24, 36, 48, 60, and 72 h post-inoculation, 300 μL of allantoic fluid was collected to determine the viral titer. The viral titer was measured as 50% egg infections dose per mL (EID_50_/mL) according to the Reed–Muench method. Viral titers were determined by three independent experiments. Meanwhile, the infected fluid was inoculated into MDCK cells. After a 1 h adsorption period, the cells were washed with PBS and supplemented with TPCK medium. The cell supernatant was then collected every 12 h and inoculated into 9-day-old SPF chicken embryos. The viral titer was determined 72 h post-inoculation.

### 2.6 The thermal stability of strain

To determine the thermal stability of the strain, the integrity of HA protein was evaluated by the HA hemagglutination test. The virus solution was diluted to 7 log2 with sterile PBS and placed in a PCR instrument. Two experimental conditions were tested: (1) incubation at different temperatures (50 °C, 52 °C, 54 °C, 56 °C, 58 °C, 60 °C, and 62 °C) for 45 min each, and (2) incubation at 56 °C for varying durations (5, 10, 15, 30, 60, 90, 120, 150, 180, and 240 min). After cooling, the HA titer was determined using 1% chicken red blood cell suspension.

### 2.7 The pathogenicity of strain to mice

Six-week-old female Balb/c mice were divided into the challenge group and the control group, with 10 mice in each group. Following ether anesthesia, mice in the challenge group were intranasally inoculated with 50 μL of the virus strain at a concentration of 10^6^ EID₅₀/mL, mice in the control group were intranasally inoculated with 50 μL of PBS. The mice were monitored daily for clinical signs, changes in body weight and mortality for 7 consecutive days.

1. Detection of body weight changes and blood virus titers in mice after challenge: after ether anesthesia, mice were intranasally inoculated with 50 μL of the virus dilution at a concentration of 10^6^ EID₅₀/mL, with 5 mice per group. Body weight and mortality were recorded daily for 7 consecutive days.
2. Detection of virus content in tissues: at 1, 3, 5, and 7 days post-infection (DPI), three mice were randomly sacrificed. The heart, liver, spleen, lung, kidney, brain and other tissues of the mice were aseptically collected, then used to inoculate 9-day-old SPF chicken embryos to detect viral load, which was measured as EID_50_/mL according to the Reed–Muench method, and to investigate the infection characteristics and *in vivo* distribution of the isolated strain.
3. Histopathological observation and immunohistochemical staining of the bone tissues of the lungs and turbinates in mice: three mice were randomly sacrificed at 3 DPI and 5 DPI. The tissues were taken and fixed in a 10% formaldehyde solution to make paraffin sections. Then stained with hematoxylin–eosin (HE) and immunohistochemistry for histopathological observation. Immunohistochemical staining was performed using mouse anti-influenza NP monoclonal antibody at a dilution of 1:800, and the secondary antibody was HRP-labeled goat anti-mouse antibody. DAB color development was adopted and observation was conducted under a microscope.

### 2.8 The transmission ability of strain among guinea pigs

Nine healthy guinea pigs were intranasally inoculated with 10^6^ EID_50_/mL and were randomly divided into three groups (challenge group, direct contact group and aerosol seeding group). Nasal aspirates were collected at 1, 3, and 5 DPI and serially diluted from 10^−1^ to 10^−4^. The diluted samples were then inoculated into 9-day-old SPF chicken embryos, and the transmission ability of the virus was assessed 72 h later.

### 2.9 Data analysis

The graph was plotted using GraphPad Prism 5.0 software. The virus titers in mouse tissues and the proliferation ability on chicken embryos were statistically analyzed by SPSS 20 software. The statistical significance of the differences between groups was determined by *t*-test. *p* < 0.05 indicated a significant difference and *p* < 0.01 indicated an extremely significant difference.

## 3 Results

### 3.1 Homology and phylogenetic analysis of HA

**Figure 1:**
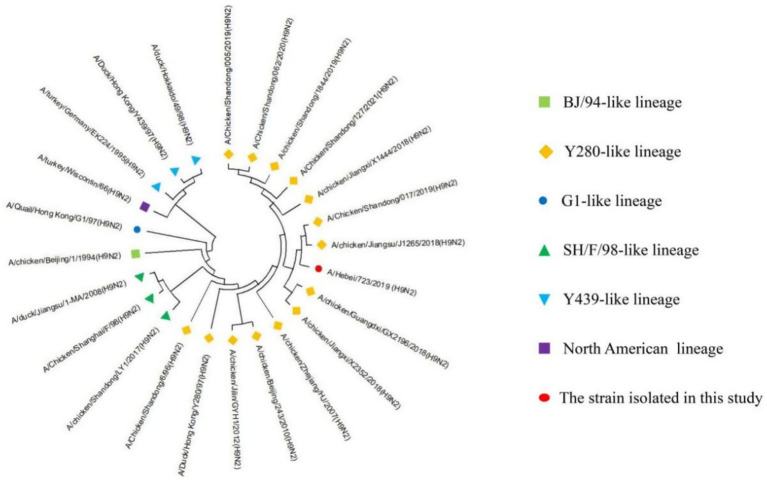
Homology and phylogenetic analysis of the HA genes of H9-subtype AIV strains. The red circle represents the strain isolated in our current study; other reference strain sequences were downloaded from GenBank. Strain lineages are shown at right.

The nucleotide homology (Table 2) of the HA gene of A/Hebei/723/2019 (H9N2) was the highest with the Y280-like lineage, ranging from 92.6 to 98.6%, and the lowest with the North American lineage (76.4%). The homology with BJ-94-like, G1-like, SH/F/98-like, and Y439-like was 85.7, 81.5, 84.6% ~ 87.5, and 82.8% ~ 85.3%, respectively. The genetic evolution analysis and genetic evolution tree were established through MEGA11, and the results showed that the HA gene of A/Hebei/723/2019 (H9N2) belongs to the Y280-like lineage of the Eurasian lineage (Figure 1).

**Table 2:** Nucleotide homogenous analyses of H9-subtype AIV isolate with reference strains.

Gene	Nucleotide length	Nucleotide sequence homology (%)					
		BJ/94-like	Y280-like	G1-like	SH/F/98-like	Y439-like	North American
HA	1,611	85.7	92.6 ~ 98.6	81.5	84.6 ~ 87.5	82.8 ~ 85.3	76.4
NA	1,299	86.3	94.2 ~ 98.3	83.6 ~ 85.2	\	84.7 ~ 85.8	76.5 ~ 77.8
PB2	2,244	84.7 ~ 85.8	86.1 ~ 89.4	95.7 ~ 99.3	85.5 ~ 89.2	87.6 ~ 90.4	74.2 ~ 78.9
PB1	2,205	88.3 ~ 90.5	89.2 ~ 91.4	87.6 ~ 90.8	90.6 ~ 92.5	98.7	77.7 ~ 79.4
PA	2034	85.2 ~ 87.4	\	87.3 ~ 89.1	94.7 ~ 96.9	88.5 ~ 89.6	74.6 ~ 78.3
NP	1,461	90.1 ~ 92.7	85.8 ~ 89.3	86.1 ~ 88.5	95.4 ~ 97.8	87.1 ~ 88.7	77.2 ~ 78.5
M	918	91.8 ~ 92.6	96.1 ~ 98.5	89.3 ~ 90.5	90.8	92.1 ~ 93.4	75.4 ~ 80.6
NS	861	86.5 ~ 90.3	\	89.2 ~ 91.3	96.4 ~ 99.2	90.2 ~ 93.7	72.7 ~ 79.1

Further comparison revealed that the HA protein cleavage site of the A/Hebei/723/2019 strain was PSRSSR↓GL, which was different from the two reference strains, the vaccine strain A/chicken/Shanghai/F/98 (referred to as SH/F/98 strain) and the classic strain. At position 334, A was replaced by S, still maintaining a non-continuous acidic state, which was in line with the typical LPAIV molecular characteristics. The amino acid sequence of the receptor binding site on the right side (positions 146–150) of the A/Hebei/723/2019 strain was the same as that of the SH/F/98 strain, which was GTSKA; compared with the Qu/G1/97 strain, the 149th position changed from K to R. The amino acid sequence on the left side of the A/Hebei/723/2019 strain was NGLMGR, which differed from that of the Qu/G1/97 strain and the SH/F/98 strain. The receptor binding site was LMG, indicating a preference for specific receptor binding with human *α*-2,6 receptor (Table 3).

**Table 3:** Comparison of HA receptor binding sites and amino acid cleavage sites.

Strains	The receptor binding site on the left side (positions 232–237)	The receptor binding site on the right side (positions 146–150)	Amino acid cleavage site (positions 333–340)
SH/F/98	NGQQGR	GTSKA	PARSSR↓GL
Qu/G1/97	NDLQGR	GTSRA	PARSSR↓GL
A/Hebei/723/2019	NGLMGR	GTSKA	PSRSSR↓GL

The results of the HA receptor binding key site of the A/Hebei/723/2019 (H9N2) strain showed that compared with the SH/F/98 strain, mutations occurred at M107L, S183N, A198T, M224L, and Q234L; compared with the Qu/G1/97 strain, mutations occurred at T107L, S183N, H191N, E198T, and V224L (Table 4).

**Table 4:** Comparison of HA receptor binding key points.

Strains	100	107	161	163	183	191	198	202	203	224	226	234 ~ 236
SH/F/98	R	M	W	T	S	N	A	L	Y	M	G	QQG
Qu/G1/97	R	T	W	T	S	H	E	L	Y	V	G	LQG
A/Hebei/723/2019	R	L	W	T	N	N	T	L	Y	L	G	LMG

### 3.2 Homology and phylogenetic analysis of NA

The nucleotide homology (Table 2) of the NA gene of A/Hebei/723/2019 (H9N2) was the highest with the Y280-like lineage, ranging from 94.2 to 98.3%, and the lowest with the North American lineage, ranging from 76.5 to 77.8%. The homology with BJ-94-like, G1-like, and Y439-like was 86.3, 83.6% ~ 85.2, and 84.7% ~ 85.8%, respectively.

**Figure 2:**
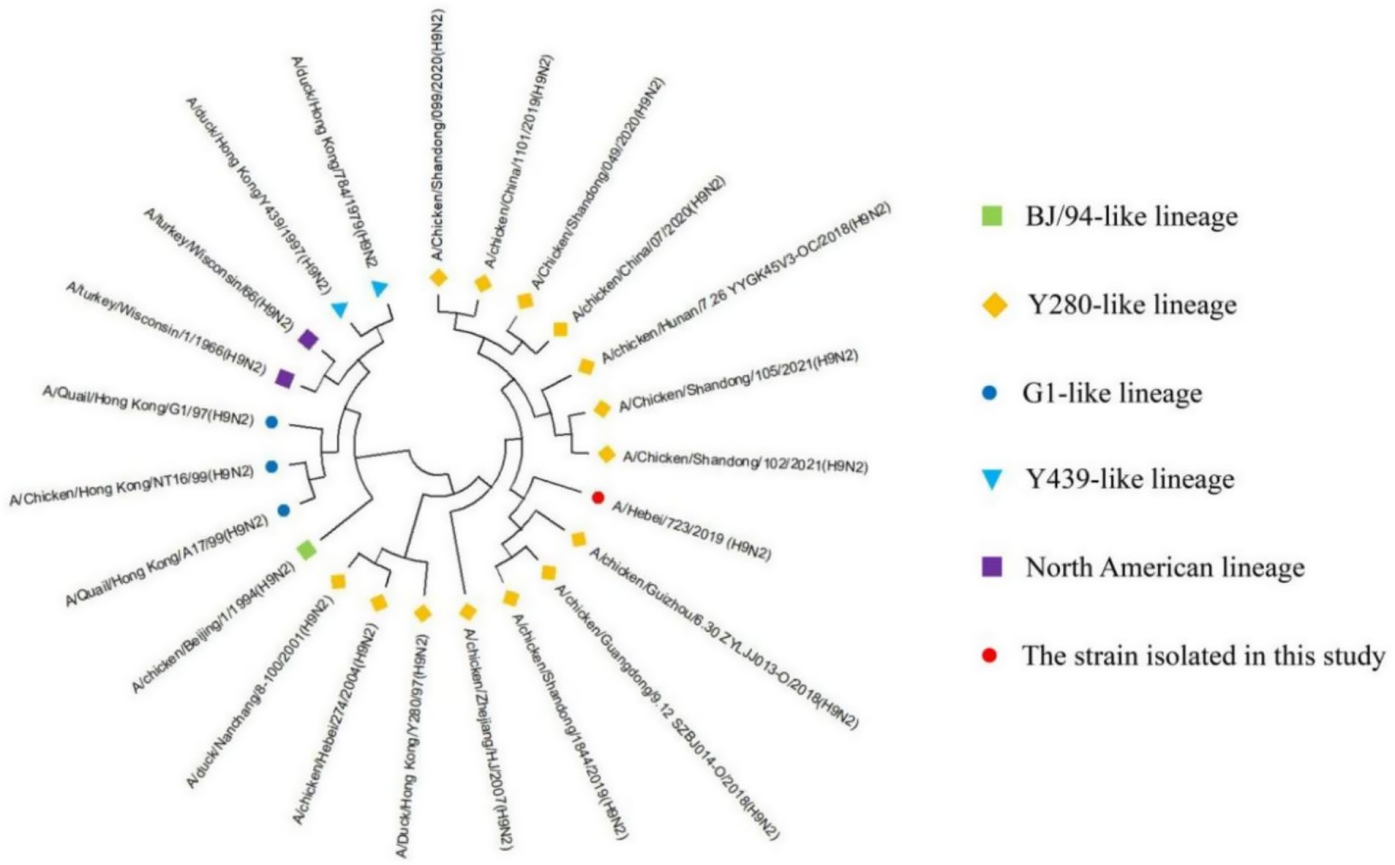
Homology and phylogenetic analysis of the NA genes of H9-subtype AIV strains. The red circle represents the strain isolated in our current study; other reference strain sequences were downloaded from GenBank. Strain lineages are shown at right.

The genetic evolution analysis and genetic evolution tree were established through MEGA11, and the results showed that the NA gene of A/Hebei/723/2019 (H9N2) belongs to the Y280-like lineage of the Eurasian lineage (Figure 2). The 63–65 positions of the amino acids that enhance the virulence were not missing, and the E119G related to the resistance to neuraminidase inhibitors did not undergo any variation; the amino acid was T.

### 3.3 Homology and phylogenetic analysis of six internal gene segments

The nucleotide homology results of the A/Hebei/723/2019 (H9N2) internal gene segments (Table 2) showed that the PB2 gene was the highest with the G1-like lineage, ranging from 95.7 to 99.3%; the PB1 gene was the highest with the Y439-like lineage (98.7%); the PA gene was the highest with the SH/F/98-like lineage, ranging from 94.7 to 96.9%; the NP gene was the highest with the SH/F/98-like lineage, ranging from 95.4 to 97.8%; the M gene was the highest with the Y280-like lineage, ranging from 96.1 to 98.5%; the NS gene was the highest with the SH/F/98-like lineage, ranging from 96.4 to 99.2%. However, all six internal gene segments were the lowest with the North American lineage.

**Figure 3:**
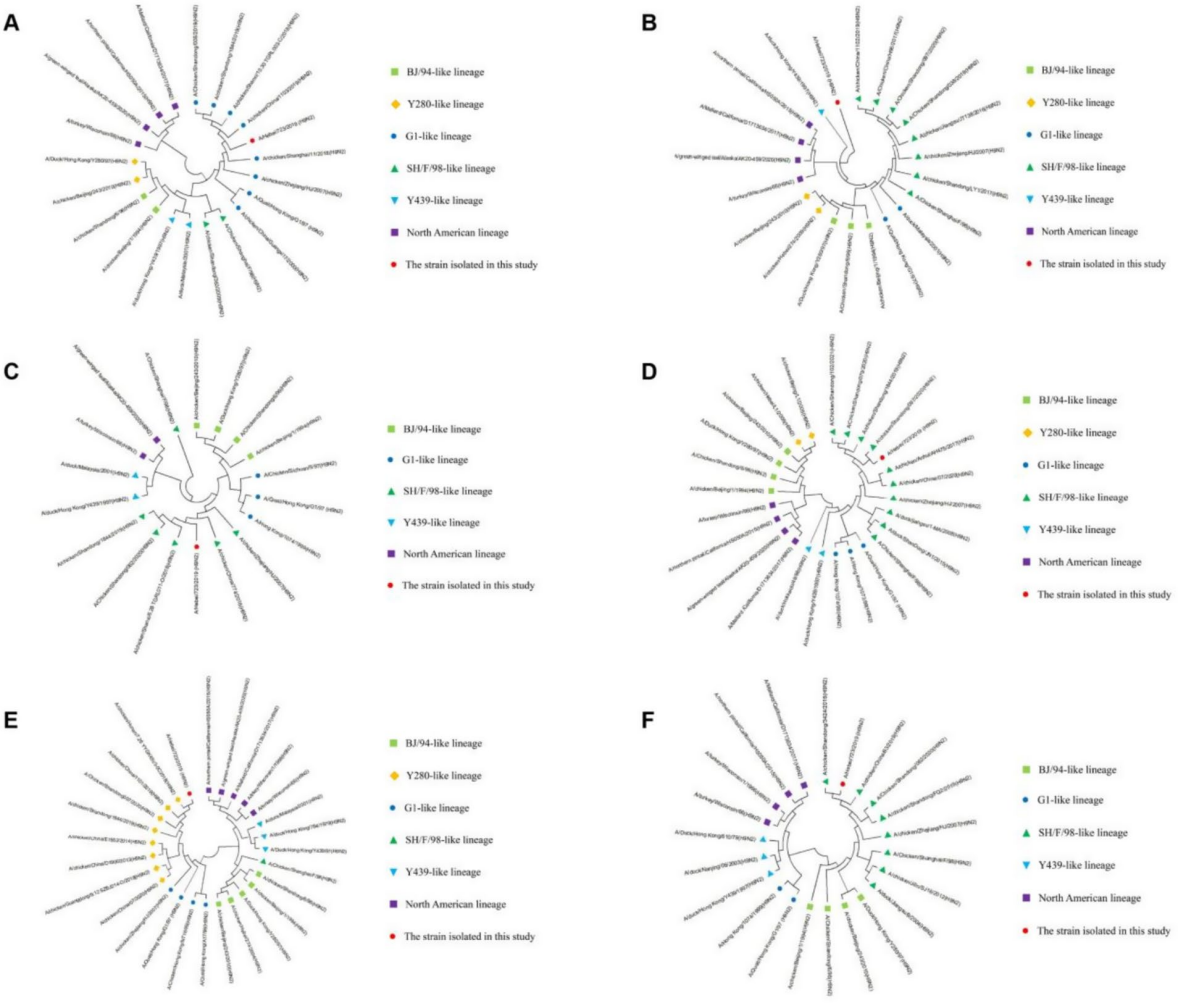
Homology and phylogenetic analysis of six internal gene segments of H9-subtype AIV strains. Homology and phylogenetic analysis of the PB2 genes **(A)**, the PB1 genes **(B)**, the PA genes **(C)**, the NP genes **(D)**, the M genes **(E)**, and the NS genes **(F)** of H9-subtype AIV strains. The red circle represents the strain isolated in our current study; other reference strain sequences were downloaded from GenBank. Strain lineages are shown at right.

The genetic evolution analysis and genetic evolution trees of A/Hebei/723/2019 (H9N2) were established through MEGA11, and the results showed that the PB2 gene belongs to the G1-like lineage of the Eurasian lineage (Figure 3A), no mutations were found in the T495V that enhances the polymerase activity and the K702R related to the host specificity transformation; the amino acids are L and S, respectively. The PB1 gene belongs to the Y439-like lineage of the Eurasian lineage (Figure 3B), no mutations occurred at the N66S amino acid site, which is related to the enhanced virulence in mice, nor at the A3V and H436Y sites, which are related to the enhanced virulence in mammals; all the amino acids were A. The PA gene belongs to the SH/F/98-like lineage of the Eurasian lineage (Figure 3C), no variation occurred at the 55th position of the aa when compared with the classic strain SH/F/98, an A → F change occurred at the 85th position, a L → F change occurred at the 226th position, and a M change occurred at the 266th position. The NP gene belongs to the SH/F/98-like lineage of the Eurasian lineage (Figure 3D), the I33V related to the host-specific transformation did not undergo any mutation; the amino acid was R. The M gene belongs to the Y280-like lineage of the Eurasian lineage (Figure 3E), no mutations occurred at the N30D site related to enhanced virulence in mice and the S31N site related to resistance to amine-based drugs. The amino acids at these sites were S and R, respectively; the S89G site related to host-specific transformation was absent. The NS gene belongs to the SH/F/98-like lineage of the Eurasian lineage (Figure 3F), the amino acid sequence analysis revealed that the NS gene of the A/Hebei/723/2019 (H9N2) isolate encodes 286 amino acids, with positions 80 to 84 not missing; the amino acid sequence is AGACC.

### 3.4 The growth dynamic curve of chicken embryos and MDCK cells

**Figure 4:**
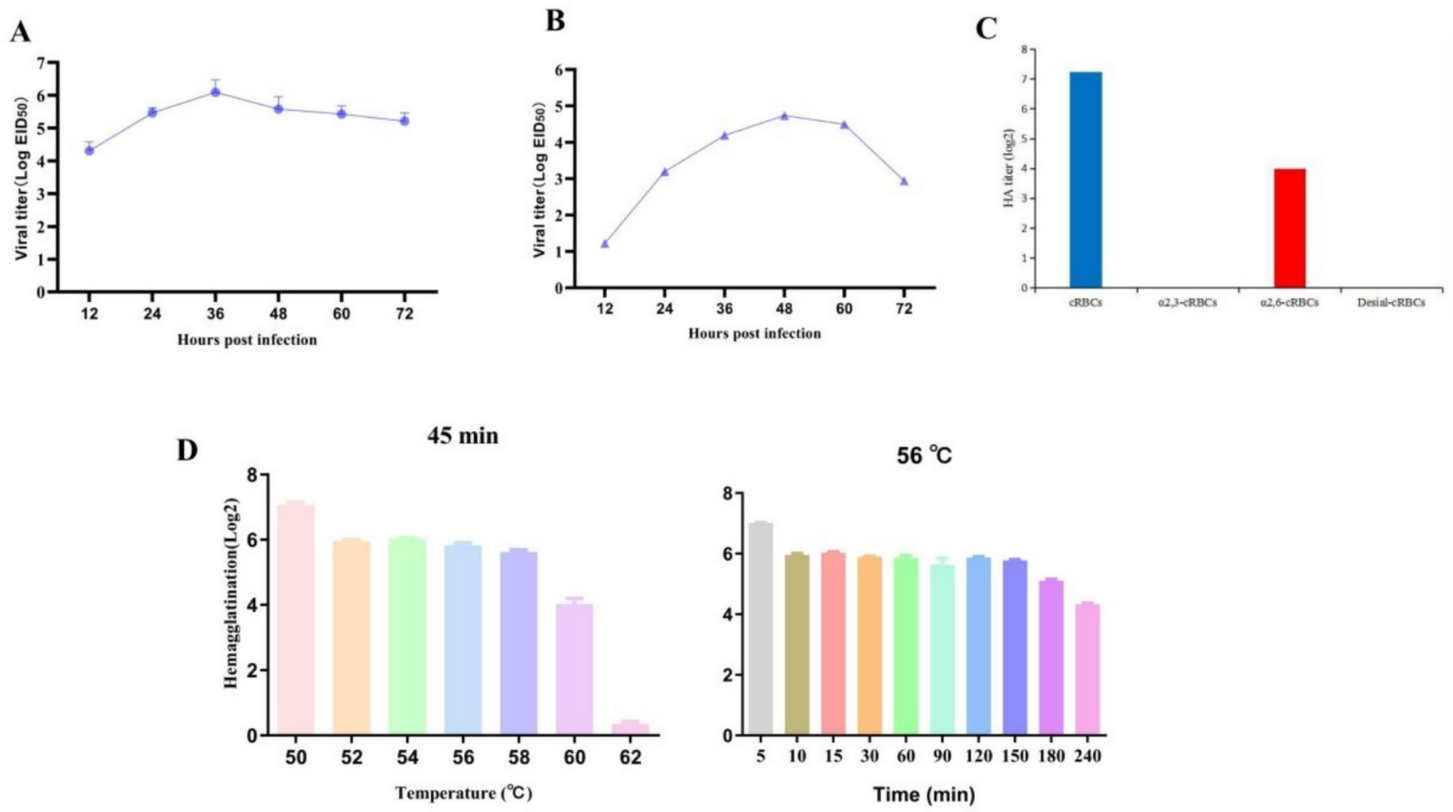
The growth dynamic curve in chicken embryos and MDCK cells, the receptor binding characteristics and HA thermal stability of the isolated strain. **(A)** The growth curve of the isolated strain on SPF chiken embryo. **(B)** The growth curve of the isolated strain on MDCK cells. **(C)** Receptor binding capacity of the isolated strain. **(D)** The results of HA thermal stability.

The EID_50_ determination result of A/Hebei/723/2019 (H9N2) was 10^8.5^ EID_50_/mL, indicating that the strain has a good replication ability on chicken embryos. After the isolated strain was inoculated into 9-day-old SPF chicken embryos, the viral titer reached the highest at 36 h, with a virus titer of 10 ^6.09^ EID_50_/mL, and the viral titer was the lowest at 12 h, at 10^4.31^ EID_50_/mL, showing a trend of first increasing and then decreasing (Figure 4A). In addition, the viral titer in MDCK cells gradually increased and then decreased, reaching the peak at 48 h, with a virus titer of 10^4.53^ EID_50_/mL (Figure 4B).

### 3.5 The receptor binding characteristics and HA thermal stability of strain A/Hebei/723/2019 (H9N2)

The receptor binding characteristics results showed that the environmental source A/Hebei/723/2019 (H9N2) had the ability to bind to the *α*-2, 6-sialic acid receptor, but not to the α-2, 3-sialic acid receptor (Figure 4C).

When the virus was incubated at a series of high temperatures (50 °C-62 °C) for 45 min, the hemagglutination titer gradually decreased, with the highest hemagglutination titer at 50 °C. At 52 °C-60 °C, there was no significant change. After incubation at 62 °C, the blood coagulation ability was basically lost, showing that the HA thermal stability is good. The persistence of thermal stability was further assessed, and the results showed that the viral titer decreased within 4 h. The change in blood coagulation titer was relatively small from 10 min to 150 min of incubation, and slightly decreased after 180 min of incubation. After 240 min of incubation, the virus still had a blood coagulation titer of more than 4log2, indicating that this strain has a strong continuous infection ability at a certain temperature (Figure 4D).

### 3.6 The isolates are pathogenic to mice

**Figure 5:**
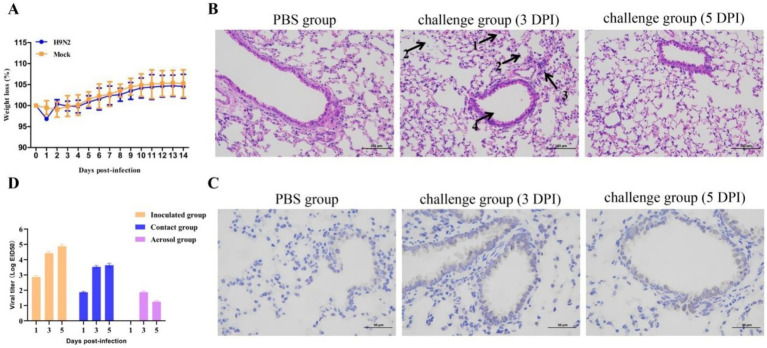
The pathogenicity and transmission capacity analysis of the isolated strain. **(A)** The body weight changes of mice after-infection. **(B)** The result of histopathological observation of lungs: the alveolar walls were congested and hemorrhagic (arrow 1); the epithelial cells of the alveolar wall partially shed, and the number of macrophages increased (arrow 2); focal widening of the alveolar walls and lymphocyte infiltration (arrow 3); partial necrosis and shedding of the mucosa of the bronchioles (arrow 4). **(C)** The result of lungs staining by IHC. **(D)** The transmission ability of isolated strain among guinea pigs.

During the experiment, none of the mice died and the mortality rate was 0%. The body weight of the blank control group increased gradually, while the challenge group presented clinical symptoms such as listlessness, loss of appetite, reduced activity, and a slight decrease in body weight (Figure 5A).

The virus was detected in all the aforementioned tissues at 3 DPI after virus challenge. Among them, the virus titer was highest in the lungs, which was 10^5.95^ EID_50_/mL. At 5 DPI and 7 DPI, viral presence was limited to the heart and lungs (*p* < 0.05). The viral titers in the heart remained at 10^3.2^ EID₅₀/mL, while the lung titers were higher, at 10^5.25^ EID₅₀/mL on both days (Table 5).

**Table 5:** Distribution of tissue virus in mice infected with A/Hebei/723/2019 (H9N2) strain.

DPI	Heart	Liver	Spleen	Lung	Kidney	Brain
3	3.95 ± 0.16	1.95 ± 0.16	3.95 ± 0.27	5.95 ± 0.27	2.2 ± 0.16	2.95 ± 0.42
5	3.2 ± 0.27^a^	——	——	5.25 ± 0.42^a^	——	——
7	3.2 ± 0.16^a^	——	——	5.25 ± 0.27^a^	——	——

The viral titers are presented as log10 values. Compared with 3 DPI, ^a^*P* < 0.05.

### 3.7 The histopathological and immunohistochemical staining observation of mice after challenge

After challenge, mild pathological changes occurred in the lungs at 3 DPI (Figure 5B), and the alveolar walls were congested and hemorrhagic (arrow 1); the epithelial cells of the alveolar wall partially shed, and the number of macrophages increased (arrow 2); focal widening of the alveolar walls and lymphocyte infiltration (arrow 3); partial necrosis and shedding of the mucosa of the bronchioles (arrow 4). However, no obvious lesions were found in the lungs at 5 DPI after challenge. In addition, after challenge, viral antigens could be detected in the lung tissues of mice at 3 DPI and 5 DPI, mainly existing in the alveolar and bronchiolar epithelial cells (Figure 5C).

### 3.8 A/Hebei/723/2019 (H9N2) strain’s transmissible ability among guinea pigs

The virus could be detected in the challenge group and the direct contact group at 1 DPI, 3 DPI and 5 DPI, and the virus titers were 10^2.87^ EID_50_/mL-10^4.87^ EID_50_/mL and 10^1.87^ EID_50_/mL-10^3.64^ EID_50_/mL, respectively. In the aerosol transmission group, the virus was detected only at 3 DPI and 5 DPI, with the virus titers of 10^1.87^ EID_50_/mL and 10^1.25^ EID_50_/mL (Figure 5D).

## 4 Discussion

H9-subtype AIV has been continuously spreading and continuously evolving and has become the most widespread subtype in poultry in China, seriously endangering the economic status and safety of the poultry industry (24). With the H9-subtype AIV infection, poultry and laying hens show mild respiratory disease symptoms, decreased egg production rates, and soft-shelled eggs, and are even co-infected with other diseases, often resulting in death, which causes serious economic losses to the poultry industry and is one of the important diseases threatening the poultry industry in China (25, 26). In addition, the H9-subtype can also provide some or all of the internal genes for the new AIV subtypes (27–29).

The AIV genome consists of eight independent vRNA-encoding PB2 protein (alkaline phosphatase), PB1 protein, PA protein (nuclear protein), HA protein (hemagglutinin), NP protein (nuclear protein), NA protein (neuraminidase), M protein (matrix protein), and NS (non-structural protein) protein (1). PB2, PB1, and PA proteins constitute the viral polymerase complex, which mainly plays a role in viral replication and transcription (30). PB2 protein is not only an important factor in the pathogenicity of influenza virus to hosts but also related to its transmissibility (31). PB1 protein plays a key role in viral genome initiation transcription and replication (32). M protein is encoded by the vRNA7 fragment and includes two proteins, M1 and M2. M1 protein is located below the viral envelope and plays a key role in post-translational replication, packaging, and morphogenesis in viral cells (33). M2 protein is located on the surface of the virus envelope and plays an important role in many aspects of virus replication, including virus particle packaging and budding (34). NP protein fragment is encoded by vRNA5 and has the function of wrapping virus particles (35). Hemagglutinin HA and neuraminidase NA, encoded by vRNA4 and vRNA6 fragments, respectively, are two major glycoproteins that mediate the entry of viruses into host cells and promote the release of mature newborn virions from the cell surface (36). Based on the different nucleoproteins and matrix proteins of the virus, the surface hemagglutinin HA is divided into 18 subtypes, and the neuraminidase NA is divided into 11 subtypes (37).

According to the eight gene fragments, AIV can be divided into two major lineages; the Eurasian lineage and the North American lineage. The Eurasian lineage can be further divided into 5 lineages, including BJ-94-like, Y280-like, G1-like, SH/F/98-like, and Y439-like (12). In this study, after molecular identification and virus isolation, an H9-subtype AIV was isolated and purified, named A/Hebei/723/2019 (H9N2). In order to understand the genetic evolution of the strain, we used the genome and the H9N2 AIV reference gene published in GenBank for homology analysis and found that the nucleotides of the eight genes of the isolated strains had the lowest homology with the North American lineage, only 72.7 to 80.6%. The nucleotides of the HA, NA, and M genes of A/Hebei/723/2019 (H9N2) had the highest homology with the Y280-like lineage, which were 92.6–98.6%, 94.2–98.3%, and 96.1–98.5%, respectively. The nucleotides of the PA, NP, and NS genes of A/Hebei/723/2019 (H9N2) had the highest homology with the SH/F/98-like lineage, which were 94.7–96.9%, 95.4–97.8%, and 96.4–99.2%, respectively. The nucleotide homology of the PB1 gene was 98.7% with the Y439-like lineage. The PB2 gene showed the highest nucleotide homology with the G1-like lineage, ranging from 95.7 to 99.3%. The genetic evolution tree was further constructed, and it was found that the HA, NA, and M genes of A/Hebei/723/2019 (H9N2) belonged to the Y280-like lineage in the Eurasian lineage; the PA, NP, and NS genes belonged to the SH/F/98-like lineage; the PB1 gene belonged to the Y439-like lineage; and the PB2 gene belonged to the G1-like lineage. Therefore, the HA, NA, and M genes of A/Hebei/723/2019 (H9N2) strain isolated in this study were derived from the Y280-like lineage; the PA, NP, and NS genes were derived from the SH/F/98-like lineage; the PB1 gene was derived from the Y439-like lineage; the PB2 gene was derived from the G1-like lineage, and a novel AIV was created by genetic rearrangement.

As a highly variable and widely spreading pathogen, the IV has always been a focus of attention in the field of public health due to its evolving host range and pathogenicity. HA is one of the main glycoproteins on the envelope of IV, responsible for the recognition and binding of the virus to the host cell surface receptor. The sialic acid receptor binding characteristics of HA protein play a decisive role in the host selection, infection process and cross-species transmission of IV. Sialic acid receptors are mainly divided into two types: *α*-2,3 and α-2,6, and their distribution on the surface shows species differences. Typically, human influenza viruses prefer to bind to the α-2,6 sialic acid receptor, while AIV prefers to bind to the α-2,3 sialic acid receptor (38, 39). The differences in receptor binding characteristics are an important basis for the host specificity of IV. In our study, the strain A/Hebei/723/2019 (H9N2) does not have the ability to bind to the α-2,3 sialic acid receptor. However, it has the ability to bind to the α-2,6 sialic acid receptor, indicating that A/Hebei/723/2019 (H9N2) has a potential risk of infecting mammals and even humans. Some studies have shown that the HA protein mutations E190D and G225D can cause the receptor binding property of the H1 subtype IV to change from binding to α-2,3 sialic acid to binding to α-2,6 sialic acid receptor. Similarly, the HA protein mutations Q226L and G228S also enable the H2 and H3 subtype IVs to bind to α-2,6 sialic acid receptor (39–42). Likewise, the Q226L mutation plays an important role in the receptor binding property changes of H5N1 and H7N9 subtype AIVs (43, 44). It is notable that the mutations act together on the receptor binding region of the HA protein, leading to complex changes in the receptor binding properties of the virus. Therefore, when studying the cross-species transmission mechanism of the H9N2 subtype AIV, it is necessary to comprehensively consider the mutations of multiple amino acid sites of the HA protein. In addition, the adaptive mutations of the viral polymerase complex are also of crucial importance. Some studies have shown that mutations such as E627K and D701N of PB2 protein can enhance the virus replication ability in mammalian cells, while specific mutations of proteins such as NP, PA and PB1-F2 have also been found to be related to the virus host adaptability, and can promote cross-species transmission through affecting the replication efficiency and interfering with the host’s immune response (45).

The HA thermal stability typically reflects its structural rigidity, while certain stabilization mutations (such as the formation of hydrophobic cores or disulfide bonds in the HA2 subunit) simultaneously enhance the resistance to high temperatures and low pH (46). For example, the T160A mutation of HA in H5N1 could simultaneously enhance thermal stability and acid stability. The H9N2 strain with high HA thermal stability could survive longer in the natural environment (such as contaminated drinking water, feed or feces), thereby increasing the chance of transmission among poultry or between poultry and mammals (43). H9N2 AIV has been shown to infect both pigs and humans, and its thermally stable HA protein can more easily maintain the *α*-2,6-sialic acid receptor binding conformation due to its specific receptor-binding characteristics. Furthermore, if the strain simultaneously carries mammalian adaptive mutations such as PB2-E627K, the thermal stability of HA will enhance the virus replication efficiency in the mammalian respiratory tract (47). In this study, the HA of A/Hebei/723/2019 (H9N2) strain had good thermal stability and still had persistent infection ability at a high temperature of 56 °C, suggesting that the strain more likely to survive *in vivo*. Studies have shown that the amino acids at positions 234 to 236 of the HA protein receptor binding site of H9N2 AIV are LMG, which prefers to bind to the specific receptor of human *α*-2,6 sialic acid receptor (34). Compared with the classic strain Qu/G1/97 and the vaccine strain SH/F/98, it was found that the HA protein of strain A/Hebei/723/2019 had varying degrees of variation. D94N and A263T are key receptor binding sites, and their enhanced binding ability to α-2,6 receptors is related to their virulence in mammals (48). However, the strain A/Hebei/723/2019 underwent D → R and A → L, respectively. Whether these variations affected the binding of the strain to the α − 2,6 receptor still requires further study.

In this study, 6-week-old Balb/c mice was infected through nasal drip and none exhibited significant weight loss or mortality, indicating that the strain A/Hebei/723/2019 (H9N2) had a relatively weak pathogenicity. In addition, viral titration of tissue samples revealed that the highest viral load was detected in the lungs. Histopathological observation revealed that there were no obvious pathological changes in the control group lungs, however, the challenge group lungs showed mild lesions, with congestion and bleeding of the alveolar walls, partial shedding of epithelial cells, increased macrophages, multiple focal widening, lymphocyte infiltration, and partial necrosis and shedding of the bronchiolar mucosa. The immunohistochemical results revealed that the viral antigens could be detected at both 3 DPI and 5 DPI, and were mainly present in the alveolar and bronchiolar epithelial cells of the challenge group. The amino acid sequence analysis of the A/Hebei/723/2019 (H9N2) strain in this study indicates that the D94N and A263T mutations in the HA, the deletion of the S89G site in the M protein, and the G225S mutation in the PA protein, which contribute to a certain extent to the phenomenon that this isolated strain could infect mice but does not cause their death or obvious pathological changes in the mouse experiments described in this chapter. In addition, the strain diffusion ability in different transmission routes was further evaluated through the guinea pig model, and the results showed that the strain could be detected at 1 DPI in the challenge group and the direct contact group, and the virus titer was relatively high, suggesting that direct contact is an efficient way for virus transmission, which is consistent with the transmission characteristics of most respiratory viruses. It is worth noting that the aerosol transmission group only detected low doses at 3 DPI and 5 DPI, suggesting that aerosol transmission requires accumulate to a certain virus titer in the host and poses a potential risk of spreading to mammals and even humans. Meanwhile, the virus stability in the aerosol environment is limited, and the aerosolization efficiency of respiratory secretions is time-dependent. In conclusion, the strain is more dependent on direct contact for transmission, but the aerosol transmission risk still cannot be ignored, especially in confined spaces or long-term exposure scenarios. It is necessary to further clarify the dose-effect relationship between viral load and infectivity in the future, and provide a basis for the formulation of public health prevention and control strategies.

## 5 Conclusion

In general, we analyzed the genetic evolution of surface gene and internal gene coding sequences of a strain named A/Hebei/723/2019 (H9N2), isolated in the study. The isolated and purified strain has relatively weak pathogenicity to mice. Meanwhile, the strain can be readily transmitted by close contact as well as by aerosol among guinea pigs, indicating its potential for infecting mammals. Overall, our study provides important insights for understanding the evolution of H9-subtype AIV and will contribute to future efforts to develop measures for preventing and controlling the disease.

## Data Availability

The original contributions presented in the study are publicly available. This data can be found here: Genbank repository, accession number(s) of nucleotide sequence(s): SUB15722761 723_HA PX732907, SUB15722761 723_M PX732908, SUB15722761 723_NA PX732909, SUB15722761 723_NP PX732910, SUB15722761 723_NS PX732911, SUB15722761 723_PA PX732912, SUB15722761 723_PB1 PX732913, SUB15722761 723_PB2 PX732914.
